# Metabolically healthy obesity is independently associated with 20-year incidence of cardiovascular disease: findings from the ATTICA cohort study (2002–2022)

**DOI:** 10.1038/s41366-026-02056-9

**Published:** 2026-04-18

**Authors:** Theodosios D. Filippatos, Panagiotis Katrapas, Thomas Tsiampalis, Christina Chrysohoou, Fotios Barkas, Evangelos Liberopoulos, Petros P. Sfikakis, Costas Tsioufis, Christos Pitsavos, Demosthenes Panagiotakos, the ATTICA study group, Elpiniki Vlachopoulou, Christina Vafia, Konstantina Kyrili, Ekavi N. Georgousopoulou, Agathi Ntzouvani, Dimitris Mpougatsas, Nikolaos Skourlis, Christina Papanikolaou, Georgia-Maria Kouli, Aimilia Christou, Adella Zana, Maria Ntertimani, Aikaterini Kalogeropoulou, Evangelia Pitaraki, Alexandros Laskaris, Mihail Hatzigeorgiou, Athanasios Grekas, George Dedoussis, Georgia Anastasiou, Amalia Despoina Koutsogianni, Evangelinos Michelis, Manolis Kambaxis, Kyriakos Dimitriadis, Ioannis Andrikou, Amalia Sofianidi, Natalia Sinou, Aikaterini Skandali, Christina Sousouni, Natassa Katinioti, Labros Papadimitriou, Konstantina Masoura, Spiros Vellas, Yannis Lentzas, Konstantina Palliou, Vassiliki Metaxa, Efi Tsetsekou, Carmen Vassiliadou, Marina Toutouza-Giotsa, Konstantina Tselika, Sia Poulopoulou, Maria Toutouza

**Affiliations:** 1 Department of Internal Medicine, School of Medicine, University of Crete, Heraklion, Greece; 2 Department of Nutrition and Dietetics, School of Health Sciences and Education, Harokopio University of Athens, Athens, Greece; 3 First Cardiology Clinic, Medical School, National and Kapodistrian University of Athens, Hippokration Hospital, Athens, Greece; 4 Department of Internal Medicine, School of Medicine, University of Ioannina, Ioannina, Greece; 5 First Department of Propaedeutic Internal Medicine, Medical School, National and Kapodistrian University of Athens, Laiko General Hospital, Athens, Greece; 6 School of Health Sciences and Education, Harokopio University of Athens, Athens, Greece; 7 National and Kapodistrian University of Athens, Athens, Greece

**Keywords:** Cardiovascular diseases, Obesity

## Abstract

**Background:**

Individuals with excess body weight do not share the same risk for cardiovascular disease (CVD). The phenotype of metabolically healthy obesity (MHO) has drawn the attention of the scientific community due to a potentially reduced CVD risk compared with the phenotype of metabolically unhealthy obesity (MUO).

**Methods:**

A prospective cohort study was conducted involving 3042 participants from the Attica region in Greece. Baseline demographic, clinical, and lifestyle characteristics were assessed, with participants categorized by their obesity and metabolic health status. Cox proportional hazards models were used to analyze the association between obesity/metabolic health status and 20-year CVD incidence, adjusting for relevant covariates.

**Results:**

The sample at baseline comprised 38% individuals who were metabolically healthy without obesity (MHWO), 44% individuals who were metabolically unhealthy without obesity (MUWO), 6% individuals with MHO, and 12% individuals with MUO. Over the 20-year follow-up, 718 participants experienced a CVD event; participants with MUO demonstrated the highest incidence rate (61.1%). Cox regression analyses revealed that individuals with MUO had an 85% higher risk of developing CVD compared with individuals having the MHWO phenotype (HR = 1.85, 95% CI = 1.02–3.58). Individuals with MHO had a 39% elevated risk compared with individuals having the MHWO phenotype (HR = 1.39, 95% CI = 1.06–3.42). Groups with MHO and MUO had independently increased CVD risk, even after multivariable adjustment.

**Conclusions:**

Individuals with MUO exhibit the highest risk, but individuals with MHO also have an independently increased CVD risk, emphasizing the significant impact of both obesity and metabolic health status on long-term CVD incidence.

## Introduction

Obesity is a major public health threat since it is a strong predisposing risk factor for cardiovascular disease (CVD) and is associated with numerous metabolic disorders (mainly type 2 diabetes), hypertension, respiratory and musculoskeletal disorders, certain types of cancer, and mental health issues [1, 2]. The term “metabolic syndrome” has been used to identify individuals with obesity and metabolic derangements, including increased waist circumference, blood pressure, triglycerides, glucose, and low high-density lipoprotein cholesterol (HDL-C). However, not all individuals with excess body weight share the same risk for CVD and other disorders. There may be differences in the distribution of the common demographic, lifestyle, clinical, and biochemical CVD risk factors within different subgroups of individuals with obesity that may modify the relationship between obesity and CVD outcomes. Understanding these differential effects may assist in both identifying high-risk populations and developing targeted interventions [3].

A subset of individuals with obesity is classified as having ‘metabolically healthy obesity’ (MHO), a condition in which a high body mass is accompanied by a healthy metabolic profile characterized by normal insulin sensitivity, lipid profiles, and arterial blood pressure. The term “MHO” has been used to imply a similar CVD risk compared with normal-weight individuals [4]. Although many consider MHO a relatively benign condition associated with a lower risk of CVD over time, substantial evidence indicates that it is linked to an increased incidence of CVD [5, 6]. There are still several unanswered questions and controversies whether it is an independent or a mediating/moderating CVD factor [4, 7]. This study aimed to assess the association between obesity, metabolic status, and the 20-year incidence of CVD among ATTICA Study participants, with a focus on the independent role of MHO.

## Methods

### Design

The ATTICA study was a prospective epidemiological cohort comprising three follow-up evaluations. The primary aims were to document the prevalence of socio-demographic, clinical, lifestyle, biochemical and psychological risk factors related with CVD; to explore the connections between these factors and the long-term risk of CVD;, and to assess their predictive significance for CVD patterns [8].

### Setting

The study was conducted from 2002 to 2022. Study participants were from the Attica region in Greece, with 78% residing in the capital city and urban municipalities.

### Sample

The ATTICA cohort consisted of 3042 males and females, recruited from 4056 invitees (a participation rate of 75%), all free from any evidence of CVD, malignancy, or chronic inflammatory conditions. Participants were selected via random sampling, stratified by age group, sex, and region in accordance with the 2001 Greek census. Comprehensive details regarding the objectives, design, sampling methodology, and procedures of the study have been described previously [8–10].

In 2022, among the 3042 participants, 2169 were located and participated in the follow-up examination (participation rate of 71%). Of the 873 individuals lost to follow-up, 771 were unreachable due to changes in their contact details or inaccuracies in their addresses or phone numbers, while 102 declined the participation in the follow-up examination. Detailed clinical data were collected through face-to-face interviews conducted by trained healthcare professionals and from participants' medical records. In cases where participants had died during the follow-up period, information was obtained from their next of kin and official death records. Anthropometric measurements, lifestyle habits, and behavioral traits were evaluated in a manner consistent with the baseline examination. Additionally, vital and CVD status details, as well as the occurrence of traditional CVD risk factors such as diabetes, hypercholesterolemia, and hypertension, were documented. Of the 2169 traced individuals, 1988 participants had complete data on baseline metabolic/obesity status, covariates, and CVD outcome and were therefore included in the final analytic sample. The remaining 181 were excluded due to missing information on one or more key variables (e.g., incomplete biochemical or lifestyle data, or uncertain outcome classification). Notably, fatal CVD events were included in the endpoint, and participants who died during follow-up were retained in the analysis, provided that outcome and covariate data were available.

### End-point assessment

This study focused on the incidence of combined (fatal or non-fatal) CVD events, as defined by the World Health Organization (WHO)–International Classification of Diseases (ICD)-10 criteria. For participants who had died, data were collected from death certificates and relatives. The ascertainment of the investigated outcomes, as available from participants’ clinical records, hospital discharge forms, or death certificates was based on International Coding Disease (ICD)-10th version (but it was decided also to keep both ICD-9 coding because it was used in the intermediate 5-year follow-up; no discordant cases were observed using the two different coding systems). In particular, information about participants’ health status concerned development of: (a): myocardial infarction, angina pectoris, other identified forms of ischemia ICD-9 coding (10th edition) (410–414.9, 427.2, 427.6 (I20-I25)); coronary revascularization (414.01) (i.e., coronary artery bypass surgery and percutaneous coronary intervention), (b) heart failure of different types 400.0–404.9, 427.0–427.5, 427.9, 428.- (I50.2-), and chronic arrhythmias (I49.-) (c) development of stroke (430–438 (I63.-)), as well as (d) development of hypertension, hypercholesterolemia, and diabetes (see below for diagnosis). In cases where multiple CVD events occurred, only the first event was considered as the endpoint. A total of 1988 participants with complete CVD evaluation data in follow-up assessments were included in this study. The age and sex distribution of this sample was not different compared with the baseline cohort (*p* values > 0.80). Furthermore, to assess potential selection bias, we compared baseline anthropometric and metabolic characteristics between participants included in the 20-year follow-up analysis (*n* = 1988) and those lost to follow-up (*n* = 1054). No significant differences were observed in the distribution of BMI categories (*p* = 0.081), or in the prevalence of hypertension (*p* = 0.058), hypercholesterolemia (*p* = 0.156), and elevated triglyceride levels (*p* = 0.386). These results, presented in Supplementary Table 1, suggest that the analytic sample is broadly representative of the original cohort in terms of key cardiometabolic risk factors. Follow-up examinations were performed at 5 (in 2006), 10 (in 2012) and 20 (in 2022) years after enrollment; incident CVD events and deaths were recorded on an annual basis throughout the 20-year period. In particular, information was updated via annual contact with participants or their families, review of hospital and medical records, and linkage with national death registries.

### Baseline assessment

During the baseline evaluation, a physical examination was performed by a qualified physician. A detailed medical history was documented, encompassing pre-existing cardiovascular risk factors, medication usage, and familial history of cardiometabolic ailments. In addition, baseline assessments included sociodemographic, anthropometric, clinical, lifestyle, and biochemical measurements; further details can be found in a previously published paper [8]. Standard procedures were employed to measure body weight and height, with body mass index (BMI) calculated as weight (in kg) divided by the square of height (in meters). Blood samples were obtained from the antecubital vein between 8 and 10 a.m. after a 12-h fast and abstinence from alcohol. Serum was promptly collected for lipid analysis in a laboratory adhering to the World Health Organization Lipid Reference Laboratories’ standards. Measurements included serum total cholesterol, HDL-C, triglycerides, with non-HDL cholesterol calculated as the difference between total cholesterol and HDL-C. Low-density lipoprotein cholesterol (LDL-C) was computed using the Friedewald formula. Clinical characteristics such as hypercholesterolemia, hypertension, and diabetes were identified based on predefined criteria [8]. Hypertension was diagnosed when systolic blood pressure (SBP) of 140 mmHg or higher and/or a diastolic blood pressure (DBP) of 90 mmHg or higher were measured. Dyslipidemia was defined as triglyceride levels of 150 mg/dL or higher and/or high-density lipoprotein cholesterol (HDL-C) levels below 40 mg/dL (1.036 mmol/L) in men and below 50 mg/dL (1.295 mmol/L) in women. Carbohydrate metabolism abnormalities were indicated by a fasting glucose level of 100 mg/dL (5.555 mmol/L) or higher. Additionally, the use of specific medication served as alternative indicators of these conditions. Adherence to the Mediterranean diet was evaluated using the MedDietScore (range 0–55), a diet score assessing 11 key components of the Mediterranean diet, i.e., olive oil, non-refined cereals, potatoes, fruits, vegetables, legumes, full-fat dairy products, fish, red meat, poultry, and alcohol [11]. Higher values of the score indicate greater adherence to the Mediterranean diet. Smoking status, including current, former, and never smokers, as well as pack-years of smoking, were also recorded [8].

### Definition of obesity status and metabolic health

The classification of weight status was made as follows: Normal weight was defined as having a BMI ranging from 18.5 to 25 kg/m²; overweight as having a BMI ranging from 25 to 29.9 kg/m²; obesity as having a BMI of 30 kg/m² or higher; underweight as having a BMI less than 18.5 kg/m². Metabolic status was determined according to the criteria outlined by Lavie and colleagues [12]. Specifically, a healthy metabolic status was characterized by the absence of metabolic syndrome features (except increased waist circumference for the baseline classification), including hypertension (blood pressure >140/90 mmHg or antihypertensive treatment), dyslipidaemia (i.e fasting triglycerides >150 mg/dl (>1.7 mmol/L) and/or HDL-C < 50 mg/dl (<1.30 mmol/L) for women and <40 mg/dl (<1.04 mmol/L) for men, or hypolipidemic treatment), and glycaemic abnormalities (fasting glucose >100 mg/dl (>5.6 mmol/L) or antidiabetic treatment).

Participants were categorized into four groups:

- Participants who were metabolically healthy without obesity (MHWO), defined as having BMI < 30 kg/m^2^ and healthy metabolic status.
- Participants with MHO, defined as BMI ≥ 30 kg/m^2^ and healthy metabolic status.
- Participants who were metabolically unhealthy without obesity (MUWO), defined as BMI < 30 kg/m^2^ and unhealthy metabolic status.
- Participants with Metabolically Unhealthy Obesity (MUO), defined as BMI ≥ 30 kg/m^2^ and unhealthy metabolic status.

In line with Lavie et al. [11], waist circumference was not included in the definition of metabolic health status, as it reflects body fat distribution rather than metabolic function. This allowed us to examine the role of metabolic health independently from central adiposity.

### Bioethics

The ATTICA study follows the ethical principles outlined in the Declaration of Helsinki and has received approval from the Ethics Committee of the First Cardiology Department of the National and Kapodistrian University of Athens (approval number #017/01.05.2001), as well as the Ethics Committee of the Harokopio University (approval number #38/29.03.2022). Prior to participation, all individuals were fully briefed on the study’s objectives and procedures, and their informed consent was obtained in writing.

### Statistical analysis

Continuous variables were expressed as Mean (SD: Standard Deviation) values, while categorical variables were presented as relative frequencies (%). Associations between categorical variables were examined using the Pearson chi-squared test. Comparisons of mean values for normally distributed variables among the four different metabolic health statuses were conducted using one-way Analysis of Variance (ANOVA), with Bonferroni correction applied for multiple comparisons. The normality of continuous variables was assessed using P-P plots. The crude incidence of CVD was computed as the ratio of new cases to the number of subjects who participated in the 20-year follow-up. Cox proportional hazards regression models were used to investigate the association between various combinations of obesity and metabolic health status and the 20-year incidence of CVD. In the main analyses, follow-up time (time since study entry) was used as the underlying timescale, and models were adjusted for age, sex, smoking status, level of adherence to the Mediterranean diet, LDL-C, C-reactive protein (CRP), systolic blood pressure, and the HOMA-IR index. As a sensitivity analysis, Cox models were additionally re-estimated using attained age as the underlying timescale, with age at study entry as the entry time and age at CVD event or censoring as the exit time. In these models, age was not included as a covariate, because it was represented by the time scale. Results were presented as hazard ratios (HR) with corresponding 95% confidence intervals (CI). All reported *p* values were based on two-tailed hypotheses and compared to a significance level of 5%. Statistical analyses were performed using STATA version 17 (STATA Corp, College Station, Texas, USA).

## Results

### Participant characteristics at baseline

In Table 1 the basic demographic, clinical, and lifestyle characteristics of the participants categorized by their obesity and metabolic health status are presented. The sample comprised 38% metabolically healthy individuals without obesity (MHWO, mean age 39.6 ± 12.2 years, 53% females), 44% metabolically unhealthy individuals without obesity (MUWO, 47.2 ± 14.0 years, 51% females), 6% metabolically healthy individuals with obesity (MHO, 46.2 ± 12.3 years, 43% females), and 12% metabolically unhealthy individuals with obesity (MUO, 51.6 ± 12.0 years, 45% females). Due to the definition of the groups, the MUO group was older and exhibited higher levels of glucose, HOMA-IR, as well as increased SBP and DBP levels compared with the other groups. Regarding lipid profiles, participants with MUO had higher triglyceride and LDL-C levels, the lowest HDL-C levels, and the highest CRP levels. Additionally, individuals with MUO demonstrated the least adherence to the Mediterranean diet, followed by those with MHO.

**Table 1:** Baseline characteristics of the *n* = 1988 ATTICA study participants, stratified according to their obesity and metabolic health status.

	Metabolically Healthy Without Obesity (MHWO)	Metabolically Healthy Obesity (MHO)	Metabolically Unhealthy Without Obesity (MUWO)	Metabolically Unhealthy Obesity (MUO)	
*N*	744	124	873	247	*p* value
Female, %	53	43	51	45	0.055
Age, years	39.6 (12.2)	46.2 (12.3)	47.2 (14.0)	51.6 (12.0)	<0.001
Body Mass Index, kg/m^2^	24.3 (3.1)	33.6 (3.6)	25 (2.8)	33.3 (3.0)	<0.001
Waist Circumference, cm	85.1 (13.2)	108.3 (12.3)	87.9 (12.3)	106.2 (14.0)	<0.001
Waist/hip ratio	0.8 (0.1)	0.9 (0.1)	0.9 (0.1)	0.9 (0.1)	<0.001
Glucose, mg/dL	89 (17)	95 (29)	94 (24)	103 (35)	<0.001
HOMA-IR index	2.8 (1.2)	3.3 (2.9)	3.2 (1.9)	3.8 (3.1)	<0.001
SBP, mmHg	117 (16)	129 (17)	124 (18)	136 (19)	<0.001
DBP, mmHg	6 (11)	84 (10)	79 (11)	87 (11)	<0.001
Triglycerides, mg/dL	98 (70)	134 (75)	126 (99)	155 (84)	<0.001
HDL-C, mg/dL	51 (15)	44 (11)	49 (13)	45 (17)	<0.001
LDL-C, mg/dL	112 (36)	118 (31)	129 (37)	136 (38)	<0.001
CRP, mg/dL	1.6 (2.1)	3.6 (3.4)	1.7 (2.2)	3.1 (3.1)	<0.001
Smoking ever (%)	53	59	57	56	0.415
MedDietScore, (0–55)	27.5 (6.5)	23.4 (4.8)	25.9 (5.7)	22.7 (6.8)	<0.001

Notes: Data are presented as mean (standard deviation) for continuous variables. P-values were obtained using one-way analysis of variance in case of continuous characteristics, and Pearson chi-squared test in case of categorical characteristics.

MHWO: BMI < 30 kg/m^2^ with metabolically healthy status; MHO: BMI ≥ 30 kg/m^2^ with metabolically healthy status; MUWO: BMI < 30 kg/m^2^ without metabolically healthy status; MUO: BMI ≥ 30 kg/m^2^ withοut metabolically healthy status.

Metabolically healthy status was defined as the absence of 4 metabolic syndrome components i.e., elevated triglycerides, reduced HDL-C, elevated blood pressure, and elevated fasting glucose or receiving drug treatment for any of these conditions.

To convert cm to inc, multiply by 0.3937.

To convert kg to pounds (lbs), multiply by 2.2046.

To convert triglycerides from mg/dL to mmol/L, divide by 88.57.

To convert HDL-C and LDL-C levels from mg/dL to mmol/L, divide by 38.67.

To convert CRP levels from mg/dL to mmol/L, divide by 10.

*MHWO* Metabolically Healthy Without Obesity, *MHO* Metabolically Healthy Obesity, *MUWO* Metabolically Unhealthy Without Obesity, *MUO* Metabolically Unhealthy Obesity, *SD* Standard Deviation, *SBP* Systolic Blood Pressure, *DPB* Diastolic Blood Pressure, *HDL-C* High-Density Lipoprotein cholesterol, *LDL-C* Low-Density Lipoprotein cholesterol, *CRP* = C-Reactive Protein, *HOMA-IR* homeostasis model assessment-insulin resistance.

### Obesity, metabolic health status and 20-year CVD incidence

Over the course of the 20-year follow-up period, 718 participants had a CVD event, comprising 478 cases of coronary heart disease, 26 cases of stroke, and 214 other CVD events (peripheral artery disease or combined CVD events). This equated to an overall crude CVD incidence of 36.1%, with a notable difference between genders (40% in males and 32% in females, *p* < 0.001). Among the 2169 participants reached at the 20-year follow-up, 206 were reported deceased. Of these, 96 deaths (46.6%) were attributed to cardiovascular causes. All fatal CVD events were included in the composite CVD outcome used in the analysis.

**Fig. 1:**
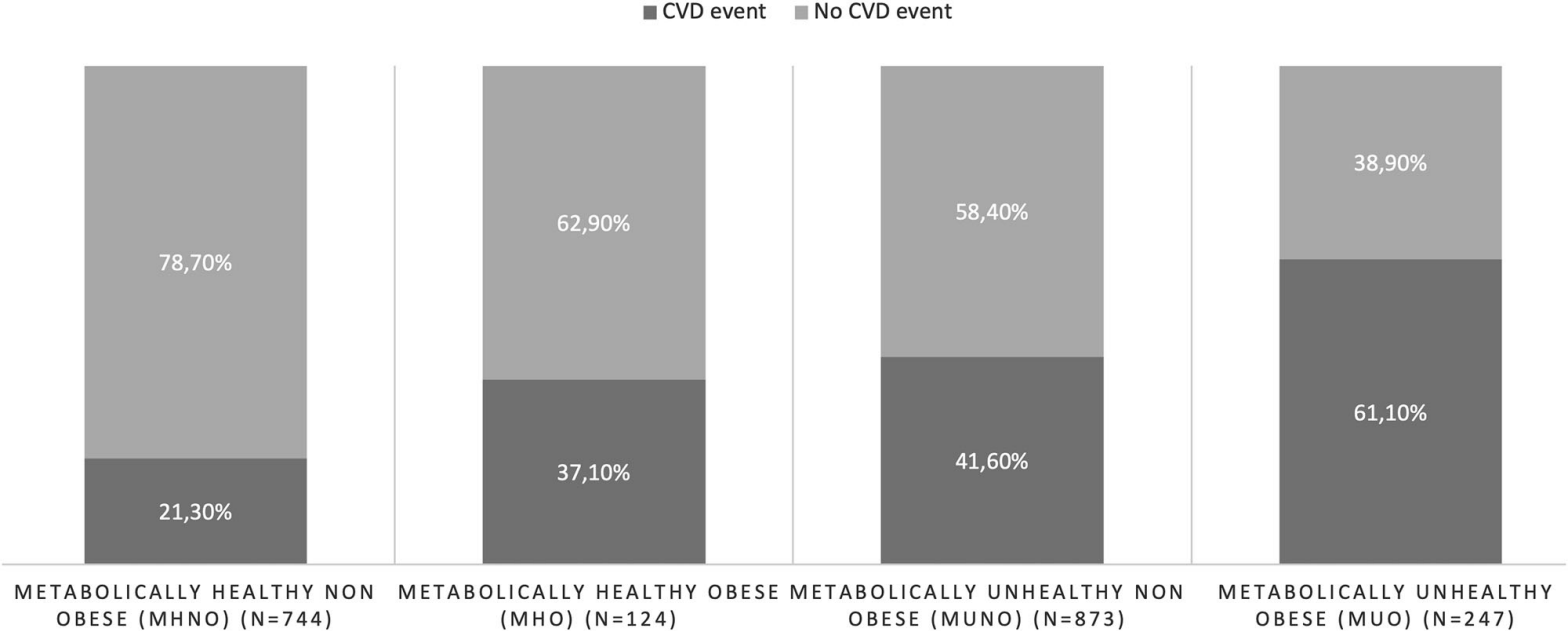
20-year incidence (%) of CVD stratified by the *N* = 1988 ATTICA study participants’ obesity and metabolic health status at baseline; ATTICA study (2002–2022).

Upon examining the impact of metabolic health status, a statistically significant contrast emerged among the four metabolic health statuses concerning the incidence of CVD over the 20-year period. Specifically, as depicted in Fig. 1, the highest incidence of CVD over 20 years was observed among participants with MUO with a rate at 61.1%, followed by participants who were MUWO at 41.6%, participants with MHO at 37.1%, and finally, participants who were MHWO at 21.3%.

The findings derived from the multivariable Cox proportional hazard analysis throughout the 20-year follow-up period are presented in Table 2. A sensitivity analysis using attained age as the underlying timescale yielded materially similar estimates (Supplementary Table 2), confirming the robustness of the primary findings.

**Table 2:** Multivariable Cox proportional hazard analyses of the association of participants’ metabolic health and obesity status at baseline with the 20-year CVD incidence; ATTICA study 2002–2022.

	MHWO	MHO	MUWO	MUO
Model 1	*Ref*	2.18 (1.46, 3.27)	2.63 (2.11, 3.28)	5.81 (4.26, 7.93)
Model 2	*Ref*	1.52 (1.02, 2.06)	1.35 (0.96, 1.88)	2.15 (1.36, 3.38)
Model 3	*Ref*	1.41 (1.11, 2.85)	1.15 (0.89, 1.92)	2.06 (1.04, 3.42)
Model 4	*Ref*	1.39 (1.06, 3.42)	1.14 (0.95, 3.27)	1.85 (1.02, 3.58)

Notes: Results are based on the multivariable Cox proportional hazards model; values are given as Hazard Ratio (95% Confidence Interval).

Models were adjusted as follows: *Model 1*: age, sex; *Model 2:* model 1 plus smoking and MedDietScore; *Model 3:* model 2 plus LDL-C, CRP, SBP; *Model 4:* model 3 plus HOMA-IR index. Metabolically healthy without obesity (MHWO): BMI < 30 kg/m^2^ with metabolically healthy status; Metabolically healthy obesity (MHO): BMI ≥ 30 kg/m^2^ without metabolically healthy status; Metabolically unhealthy without obesity (MUWO): BMI < 30 kg/m^2^ without metabolically healthy status; Metabolically unhealthy obesity (MUO): BMI ≥ 30 kg/m^2^ without metabolically healthy status. Metabolically healthy status was defined as the absence of 4 metabolic syndrome components i.e. elevated triglycerides, reduced high-density lipoprotein cholesterol, elevated blood pressure and elevated fasting glucose, including drug treatment for any of these conditions.

*MHWO* Metabolically Healthy Without Obesity, *MHO* Metabolically Healthy Obesity, *MUWO* Metabolically Unhealthy Without Obesity, *MUO* Metabolically Unhealthy Obesity, *CVD* cardiovascular disease, *LDL-C* low-density lipoprotein cholesterol, *CRP* C-reactive protein, *SBP* systolic blood pressure, *HOMA-IR* homeostasis model assessment-insulin resistance.

MUO vs. MHWO: Participants with MUO had an 85% higher risk of developing CVD compared to the MHWO. This finding is based on Model 4, which reports a hazard ratio (HR) of 1.85 and a 95% confidence interval (CI) of 1.02–3.58. Even after adjusting for several variables, the risk of CVD in the MUO group was nearly double that of the reference group.

MHO vs. MHWO: Individuals with MHO had a 39% higher risk of developing CVD over the 20-year period compared to MHWO participants. In Model 4, this is reflected by a hazard ratio of 1.39 and a 95% CI of 1.06–3.42. Even after adjustment for multiple variables, this elevated risk remained statistically significant.

MUWO group comparison: Initially, the MUWO group showed a markedly higher risk of CVD (HR = 2.63) when controlling only for age and sex. However, as additional risk factors were accounted for in Models 2 through 4, the initially observed risk diminished and lost statistical significance. This pattern suggests that the apparent elevated risk in the MUWO group in the simpler model may be largely attributed to other confounding variables rather than an independent effect of metabolic unhealthiness in the absence of obesity.

## Discussion

Obesity poses a significant public health issue due to its link with various health risks [13]. Within the population with obesity, a subgroup exists characterized as having metabolically healthy obesity (MHO), indicating a favorable metabolic profile [7]. This study provides valuable insights into the long-term implications of obesity and metabolic health status on CVD incidence. Our analyses revealed a statistically significant contrast among the four metabolic health statuses regarding CVD incidence over the 20-year observational period. The highest incidence of CVD over 20 years was observed in participants with MUO, followed by MUWO, MHO and MHWO statuses. Importantly, the results obtained from the multivariable Cox proportional hazards analyses showed that individuals classified as MHO had a 39% higher risk of experiencing CVD within the specified timeframe, which was independent of multiple variables; this independent relationship was evident only for MHO and MUO.

Obesity increases the likelihood of CVD, partially because of its close link with atherogenic dyslipidemia, i.e., increased triglycerides and low HDL-C levels [14]. The functional decline of HDL could play a role in the elevated CVD risk related with obesity, although the impact of obesity on the composition and functionality of HDL needs more evaluation [15, 16]. People with obesity have significantly lower concentration of HDL-associated free cholesterol, cholesteryl-esters, and phospholipids compared with subjects having normal weight [15]. Increased triglyceride levels, especially when combined with the presence of triglyceride-rich lipoprotein remnants and small dense LDL particles are associated with increased CVD risk [14]. In our study, in terms of lipid profiles, the participants with MUO exhibited significantly elevated levels of triglycerides and LDL-C compared with their metabolically healthy counterparts. Conversely, the MHO group showed the lowest levels of HDL-C. These lipid variables are involved in complex pathophysiological mechanisms that mediate the interplay between obesity and CVD.

Experimental and observational evidence suggest that inflammation is a key player in the pathogenesis of CVD [17]. CRP levels are positively associated with CHD risk [18, 19], although it maybe not an independent risk factor but related to several metabolic aberrations inducing inflammation of fat tissue or an ongoing atherosclerotic process. Low CRP levels among persons with MHO were associated with a CHD risk comparable with that of persons who were MHWO [20]. In our study, participants with obesity exhibited significantly increased levels of CRP compared with participants without obesity, a finding that may partly explain the association with increased CVD risk.

The results of this study are supported by previously published studies that showed a relationship between MHO and CVD risk and are consistent with relevant meta-analyses [5, 6, 21]. Our findings emphasize the importance of considering both obesity and metabolic health status when assessing CVD risk. While individuals with MHO have a better metabolic profile compared with their metabolically unhealthy counterparts, they still face an elevated CVD risk compared with individuals without obesity. Importantly, our results show an independent association of MHO and CVD risk, an observation that is not consistently reported in previous publications. It seems that the obesity-CVD association is mediated through additional mechanisms that are independent of the traditional metabolic factors. In this context, adipose tissue quality and functionality may be more important mediators of cardiometabolic risk than its total amount. Maladaptive expansion of adipose tissue has both local and systemic consequences. Locally, this expansion leads to hypoxia, impaired mitochondrial function, inflammation, and dysregulated adipokine secretion. Systemically, it contributes to a pro-inflammatory and pro-thrombotic state, endothelial dysfunction, insulin resistance, glucose/lipid metabolism dysregulation and increased blood pressure [3]. Additionally, the presence of ectopic fat on various organs dysregulates their function and is a target of current anti-obesity treatment [22–24]. Non-alcoholic fatty liver disease, aside from its potential to induce liver-related morbidity and mortality, is also linked to both subclinical and clinical CVD [25]. Epicardial fat, cardiac remodeling, and fibrosis associated with obesity may contribute to the increased incidence of CVD in subjects with obesity [26, 27]. These interconnected mechanisms serve to elucidate the strong association between obesity and CVD [3].

## Strengths and limitations

This research possesses several notable strengths. To our knowledge, the ATTICA study is the sole extensive prospective cohort study of CVD epidemiology in Greece. Moreover, it is one of the few studies globally to incorporate a prolonged and multi-phase follow-up. Furthermore, the study sample showed adequacy and representativeness concerning the age and sex distribution within the urban Greek population. A thorough evaluation of participants was conducted, encompassing clinical, biochemical, and lifestyle CVD factors. The study meticulously examined the association between MHO and the incidence of CVD over a span of 20 years, while adjusting for several risk factors and their possible mediating and moderating effects. Our report, compared with most of the previous publications, incorporates data from the first two decades of the current century, giving information on the current trends in the association of MUO and CVD risk, and has one of the longer periods of observation.

Nevertheless, this study is not without limitations. It solely relied on baseline measurements, which could result in loss of transitions owing to the considerable interval periods between follow-up assessments. The examination centered on the relationship of obesity/metabolic health status with CVD incidence in a population with generally low baseline CVD risk, as participants did not have pre-existing cardiac conditions. The loss to follow-up of 873 participants (i.e., 29%) is relatively large, but acceptable for such long-term studies. Vital status data were obtained for 2,169 individuals, so it is possible that some non-traced participants may have died during the 20-year period. However, the baseline characteristics of traced and non-traced individuals did not significantly differ with respect to BMI or metabolic risk factors, suggesting limited impact of potential mortality-related selection bias on our findings. The present study focused on baseline classification of obesity and metabolic health to preserve the prospective design and avoid survivor bias. Future analyses could explore transitions between body mass phenotypes over time and their impact on CVD risk, although such approaches would require different modeling strategies and are inherently limited to surviving participants with complete follow-up data. It is also possible that individuals classified as MHO at baseline may transition to a metabolically unhealthy phenotype as they age. While our age-adjusted models suggest an independent association between MHO and CVD, the observed younger age in this group raises the possibility that some obesity-related sequelae had not yet emerged.

## Conclusions

Metabolically healthy obesity, a term used to describe individuals with obesity but not exhibiting the typical metabolic abnormalities associated with obesity, is still a matter of debate regarding its implications on cardiovascular health. Some studies suggest that MHO individuals may have a lower risk of CVD compared to people with MUO, while others indicate that MHO individuals still face an increased risk of CVD compared with metabolically healthy individuals without obesity. The present study shows that individuals with obesity exhibit higher CVD risk compared with subjects without obesity, underlying the importance of obesity on long-term CVD risk, regardless of metabolic health status. Therefore, maintaining a healthy body weight through regular physical activity, a balanced diet, and other lifestyle modifications remains crucial for overall health and reducing the risk of chronic diseases like CVD.

## Supplementary information

Supplementary Figure 1

Supplementary Table 1

Supplementary Table 2

## Data Availability

Data described in the manuscript, code book, and analytic code will be made available upon request to the corresponding author.
