# Supplementary material for: Metabolically healthy obesity is independently associated with 20-year incidence of cardiovascular disease: findings from the ATTICA cohort study (2002–2022)
Source: Int J Obes (Lond). 2026 Apr 18;50(6):1251–7. doi: 10.1038/s41366-026-02056-9 (PMC13286986; doi:10.1038/s41366-026-02056-9)
Supplement: Supplementary file 1 — Supplementary Figure 1 [file 41366_2026_2056_MOESM1_ESM.docx]

| **Supplementary Figure 1:** Kaplan–Meier curves of CVD-free survival over 20 years, stratified by baseline metabolic/obesity phenotype |
| --- |
| 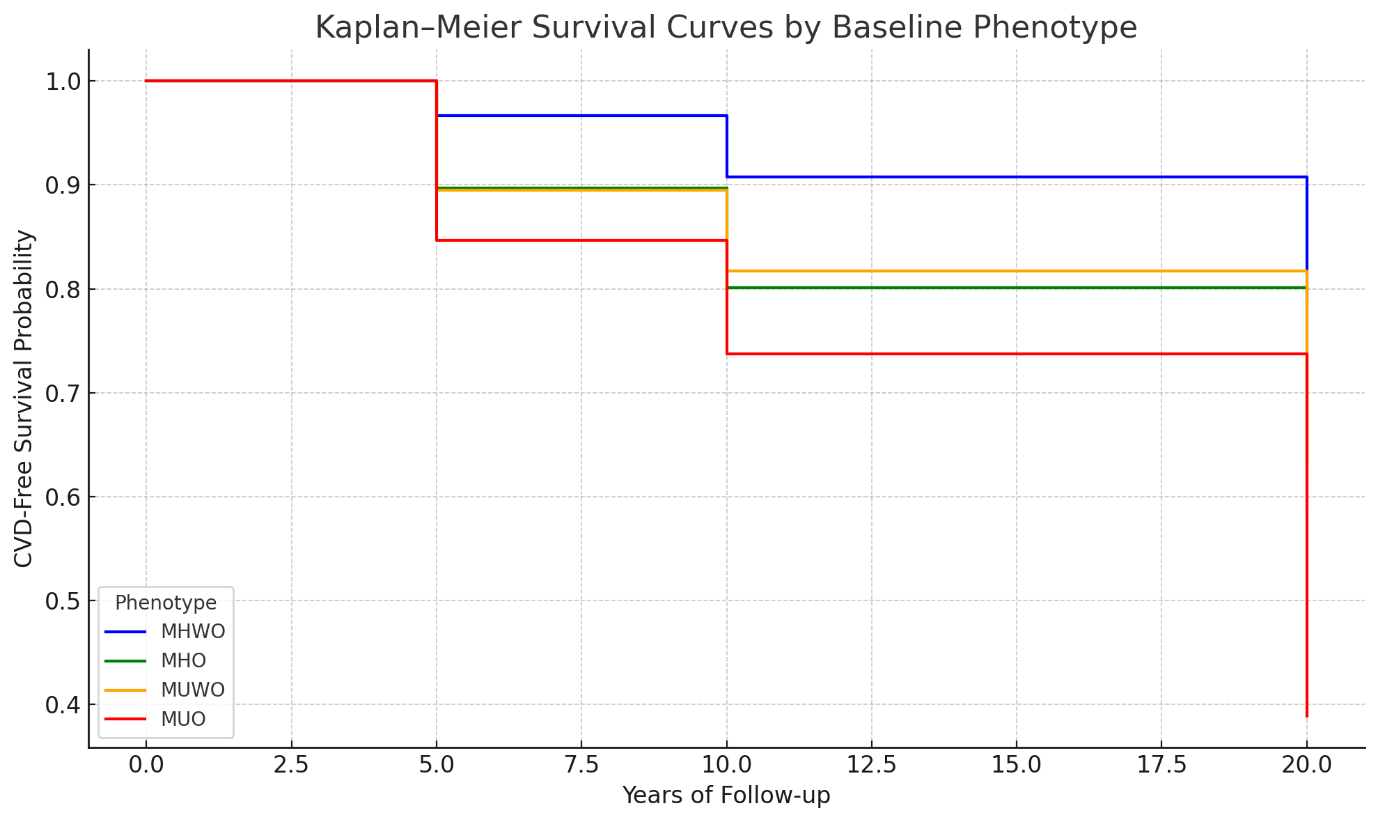 |
| Notes: The figure presents CVD-free survival probabilities among 1,988 ATTICA study participants, classified at baseline into four phenotypes: metabolically healthy without obesity (MHWO), metabolically healthy obesity (MHO), metabolically unhealthy without obesity (MUWO), and metabolically unhealthy obesity (MUO). Survival probabilities were estimated using the Kaplan–Meier method, with follow-up time defined as years from baseline to CVD event or censoring. The MUO group exhibited the lowest probability of CVD-free survival, followed by MUWO and MHO, while MHWO participants demonstrated the most favorable long-term prognosis; p-value of log-rank test< 0.001 |
