# Supplementary material for: Metabolically healthy obesity is independently associated with 20-year incidence of cardiovascular disease: findings from the ATTICA cohort study (2002–2022)
Source: Int J Obes (Lond). 2026 Apr 18;50(6):1251–7. doi: 10.1038/s41366-026-02056-9 (PMC13286986; doi:10.1038/s41366-026-02056-9)
Supplement: Supplementary file 2 — Supplementary Table 1 [file 41366_2026_2056_MOESM2_ESM.docx]

| **Supplementary Table 1.** Comparison of baseline characteristics between participants included in the 20-year follow-up analysis and those lost to follow-up in the ATTICA study (2002–2022) | | | |
| --- | --- | --- | --- |
| **Characteristic** | **Included**  **(n= 1,988)** | **Lost to follow-up**  **(n= 1,054)** | **p-value** |
| BMI category (%) |  |  |  |
| Underweight | 1.6 | 1.0 | 0.081 |
| Normal- weight | 40.4 | 36.9 |  |
| Overweight | 39.9 | 45.2 |  |
| Obesity | 18.2 | 16.8 |  |
| Hypertension (%) | 31.2 | 27.8 | 0.058 |
| Hypercholesterolaemia (%) | 42.7 | 39.7 | 0.156 |
| Elevated triglycerides levels (%) | 65.4 | 64.8 | 0.386 |
