# Supplementary material for: Metabolically healthy obesity is independently associated with 20-year incidence of cardiovascular disease: findings from the ATTICA cohort study (2002–2022)
Source: Int J Obes (Lond). 2026 Apr 18;50(6):1251–7. doi: 10.1038/s41366-026-02056-9 (PMC13286986; doi:10.1038/s41366-026-02056-9)
Supplement: Supplementary file 3 — Supplementary Table 2 [file 41366_2026_2056_MOESM3_ESM.docx]

| **Supplementary Table 2**. Hazard Ratio (HR) and 95% Confidence Interval (95%CI) for the association of participants’ metabolic health/obesity phenotype with 20-year CVD incidence, using attained age as the underlying time scale | | | | |
| --- | --- | --- | --- | --- |
|  | MHNO | MHO | MUNO | MUO |
| Model 1 | *Ref* | 2.05 (1.38–3.02) | 2.47 (1.98–3.12) | 5.39 (3.96–7.42) |
| Model 2 | *Ref* | 1.46 (1.01–2.01) | 1.29 (0.93–1.80) | 2.03 (1.29–3.21) |
| Model 3 | *Ref* | 1.38 (1.01–1.87) | 1.11 (0.82–1.64) | 1.95 (1.09–3.28) |
| Model 4 | *Ref* | 1.34 (1.01–1.80) | 1.10 (0.84–1.57) | 1.82 (1.07–3.43) |
| Notes: Results are based on multivariable Cox proportional hazards models using attained age as the underlying time scale. Models were adjusted as follows: Model 1: sex; Model 2: Model 1 plus smoking and MedDietScore; Model 3: Model 2 plus LDL-cholesterol, C-reactive protein, and systolic blood pressure; Model 4: Model 3 plus HOMA-IR index. Metabolically healthy non-obese (MHNO): BMI <30 kg/m² with metabolically healthy status; Metabolically healthy obese (MHO): BMI ≥30 kg/m² with metabolically healthy status; Metabolically unhealthy non-obese (MUNO): BMI <30 kg/m² without metabolically healthy status; Metabolically unhealthy obese (MUO): BMI ≥30 kg/m² without metabolically healthy status. Metabolically healthy status was defined as the absence of four metabolic syndrome components (elevated triglycerides, reduced HDL-C, elevated blood pressure, elevated fasting glucose or treatment for these conditions).  ***Abbreviations***: MHWO = Metabolically Healthy Without Obesity; MHO = Metabolically Healthy Obesity; MUWO = Metabolically Unhealthy Without Obesity; MUO = Metabolically Unhealthy Obesity; CVD: cardiovascular disease; LDL-C: low-density lipoprotein cholesterol; CRP: C-reactive protein; SBP: systolic blood pressure; HOMA-IR = homeostasis model assessment-insulin resistance. | | | | |
